# Population pharmacokinetics of encorafenib and binimetinib in real-world patients with BRAF^V600E/K^-mutant metastatic melanoma

**DOI:** 10.1007/s00280-026-04876-y

**Published:** 2026-03-17

**Authors:** Yuan J. Pétermann, Alicja Puszkiel, Abbas Khani, Nora Kramkimel, Elisa Funck-Brentano, Benédicte Oules, Sarah Bouchereau, Michel Vidal, Xavier Decleves, Chantal Csajka, Benoit Blanchet, Monia Guidi

**Affiliations:** 1 Centre for Research and Innovation in Clinical Pharmaceutical Sciences, Lausanne University Hospital and University of Lausanne, Rue du Bugnon 17, CH-1011 Lausanne, Switzerland; 2 Biologie du Médicament - Toxicologie, Cochin University Hospital, AP-HP, CARPEM, Paris, France; 3 Université Paris Cité, INSERM, Optimisation thérapeutique en neuropharmacologie OTEN U1144, Paris, 75006 France; 4 Neurotechnology Group, Institute of Neuroinformatics, ETH Zurich, Zurich, Switzerland; 5 Dermatology Department, Cochin University Hospital, AP-HP, Paris, France; 6 Department of General and Oncologic Dermatology, Ambroise Paré hospital, APHP, EA 4340 “Biomarkers in cancerology and hemato-oncology”, UVSQ, Université Paris-Saclay, Boulogne-Billancourt, France; 7 UMR8038 CNRS, U1268 INSERM, Faculty of Pharmacy, University Paris, PRES Sorbonne Paris Cité, Paris, France; 8 School of Pharmaceutical Sciences, University of Geneva, University of Lausanne, Lausanne, Switzerland; 9 Institute of Pharmaceutical Sciences of Western Switzerland, University of Geneva, University of Lausanne, Geneva & Lausanne, Switzerland; 10 Service of Clinical Pharmacology, Lausanne University Hospital and University of Lausanne, Lausanne, Switzerland

**Keywords:** Encorafenib, Binimetinib, Oncology, Population Pharmacokinetic, Real-world data, Therapeutic drug monitoring

## Abstract

**Purpose:**

Encorafenib and binimetinib pharmacokinetic (PK) studies in real-world cancer patients remain scarce. We aimed to characterize their population pharmacokinetics (popPK) in routine clinical care and compare real-life exposure metrics with clinical trials data.

**Methods:**

We conducted population pharmacokinetic (popPK) analyses in real-world patients with BRAF^V600E/K^-mutant metastatic melanoma to characterize interindividual variability (IIV) and identify significant covariates in MonolixSuite™. Final model-based Bayesian individual PK metrics (i.e., trough concentration, C_min_, maximal concentration, C_max_, unbound average concentration, C_average_, and area under the curve, AUC_τ_) were compared with data from clinical trials and potency-anchored metrics (reported half-maximal inhibitory concentration, IC_50_).

**Results:**

Data from 66 encorafenib and 69 binimetinib patients (310 and 311 samples) were analyzed. For both drugs, a two-compartment model with first-order absorption and linear elimination best described the data. None of the studied covariates were significantly associated with model parameters. Model-predicted PK metrics (geometric mean and CV(%)) for encorafenib and binimetinib at 450 mg QD and 45 mg BID were: C_min_ 19 ng/mL (73%) and 55 ng/mL (56%), C_max_ 3378 ng/mL (30%) and 367 ng/mL (24%), and half-lives 12.5 h (95%) and 7.3 h (67%), respectively. Encorafenib AUC_24h_ was 15,785 ng·h/mL (17%) and binimetinib AUC_12h_ was 2003 ng·h/mL (22%). Monte Carlo simulation showed that reducing encorafenib from 450 mg to 300 mg once daily lowered unbound C_average_ by 33% while maintaining substantial coverage relative to IC_50_.

**Conclusion:**

Overall, these metrics are comparable to those reported in clinical trial data. This population PK model could be useful to explore the exposure-response relationship in future PK/PD studies.

**Supplementary Information:**

The online version contains supplementary material available at https://doi.org/10.1007/s00280-026-04876-y.

## Introduction

The RAS/RAF/MEK/ERK signalling pathway plays a crucial role in various cancers, fostering the development of targeted therapies involved in that pathway [1, 2]. Following the Phase Ib/II (CMEK162X2110) and Phase III COLUMBUS study, binimetinib and encorafenib, MEK and BRAF inhibitors, respectively, received Food and Drug Administration (FDA) approval in 2018 for the treatment of metastatic or unresectable melanoma with BRAF^V600E^ or BRAF^V600K^ mutations [1, 2]. Five years later, after the Phase II PHAROS study, the drug combination was again approved by the FDA for BRAF^V600^-mutant metastatic non-small cell lung cancer, underlying the growing interest in this targeted therapies combination [3]. Following the Phase III BEACON study, encorafenib in combination with cetuximab was also approved in patients previously treated for BRAF^V600E^-mutant metastatic colorectal cancer [4].

In the treatment of metastatic melanoma, encorafenib is approved at 450 mg once daily (QD) dose in association with binimetinib 45 mg twice daily (BID). After oral administration, both encorafenib and binimetinib are rapidly absorbed with time to reach maximum concentration (T_max_) of 1.5 h and 1.1 h, respectively [5]. Encorafenib is metabolised via CYP3A4 (~ 83% of the total metabolism based on in vitro assays), CYP2C19 and CYPD26 [5], and was found to be an in vitro inducer of several enzymes including CYP3A4 [5] which probably explains the increase in its apparent clearance (CL/F) following repeated dosing [5]. The primary metabolic pathways of binimetinib include glucuronidation, N-dealkylation, and amide hydrolysis. The mean terminal half-lives of encorarenib and binimetinib are 12.1 [5, 6] and 8.7 h [6], respectively.

The pharmacokinetic-pharmacodynamic (PK/PD) relationship for encorafenib and binimetinib remains poorly described [7]. The analyses conducted by manufacturer are mainly based on dosing regimens of 300 and 450 mg QD for encorafenib and 45 mg BID for binimetinib, and revealed no statistically significant link between encorafenib exposure and efficacy [8]. Although the report suggests that an area under the curve (AUC) above the median is associated with an increased risk of grade 3 or 4 adverse events, it does not provide specific AUC values [8]. Similarly, binimetinib exhibited no association between its exposure and efficacy endpoints such as progression-free survival (PFS), overall survival (OS), or objective response rate (ORR) [8]. Moreover, findings on the exposure-toxicity relationship were conflicting, with comparable occurrences of grade 3 or 4 adverse events at AUC above and below the median but increased risk of dose reductions with AUC above the median.

In metastatic melanoma, encorafenib and binimetinib are approved at doses one level below the maximum tolerated dose (MTD) [9, 10]. These doses are used in real-world settings as the standard dosing regimen [11–14]. However, patients encountered in routine practice often differ from the highly selected patients in clinical trials, potentially leading to higher variations in plasma exposure [15]. If an exposure–response relationship for toxicity exists for encorafenib and binimetinib in real-world melanoma patients, substantial inter-individual pharmacokinetic variability in plasma exposure could increase the risk of toxicity and, consequently, dose interruptions or reductions, leading to compromised treatment efficacy [7, 16]. In this context, developing a popPK model for encorafenib and binimetinib using real-world data and identifying covariates influencing inter-individual variability (IIV) is crucial to better understand their PK in real-world populations.

In the present study, we conducted exploratory popPK analyses of plasma concentrations of encorafenib and binimetinib collected in patients with BRAF^V600E/K^-mutant metastatic melanoma and followed during routine clinical care to characterize their PK behaviour, associated inter-inidividual variability, and the influence of potential covariates.

## Patients and methods

### Studied population

Hospitalized and ambulatory cancer patients with unresectable or metastatic melanoma with BRAF^V600E^ or BRAF^V600K^ mutations under encorafenib and binimetinib for whom routine monitoring of plasma concentrations was performed at Cochin University Hospital (Paris, France) and Ambroise Paré University Hospital (Boulogne Billancourt, France) between 2018 and 2024 were retrospectively enrolled in this study. Monitoring of plasma concentrations was performed in all patients as part of routine care. Last dose intake and comedications along with demographic data including age, body weight, height, sex and relevant biological data such as bilirubin or alanine aminotransferase concentrations were collected at each sampling time point. This study was conducted in accordance with the 2008 Declaration of Helsinki. It was approved by the local ethics committee in Oncology (CLEP number: AAA-2025–10006). Data with unreliable time and/or date of last drug intake and/or sampling were excluded.

### Analytical method

Encorafenib and binimetinib plasma concentrations were measured by high-performance liquid chromatography coupled with tandem mass spectrometry (LC-MS/MS) in both hospitals. More details concerning the analytical method used in Cochin University Hospital (Paris, France) are available in Supplemental Material 1. The lower limit of quantification (LLOQ) was 1 ng/mL for both drugs. Plasma samples from patients followed in Ambroise Paré University Hospital were locally assayed using LC-MS/MS method, with the same LLOQs for both drugs. The accuracy of both methods was ensured by the external quality assessment scheme provided by the Group of Clinical Pharmacology in Oncology (Unicancer, Paris, France) and Asqualab (Paris, France).

### Population pharmacokinetic analysis

PopPK analyses were performed using the MonolixSuite™ including Monolix, Sycomore and Simulx version 2024R1. Statistical analysis, data management and graphical exploration were performed using R version 4.2.2. As all samples were collected at least 25 days after treatment initiation or modification of the dosing regimen, enrolled patients were assumed to have reached steady-state, given the reported auto-induction time of 15 days of encorafenib [7]. Two encorafenib and five binimetinib samples below the LLOQ were excluded from the analyses.

### Base and covariate models

The best model was identified using a classical stepwise procedure, comparing models with an increasing number of compartments, assuming linear elimination and absorption. Due to insufficient data covering the absorption phase, models for both drugs were built fixing the values of first-order absorption rates constants (ka). Selection of the ka was based on the relevance of the estimated T_max_ compared to the literature range of 0.5–2.6 h for both encorafenib and binimetinb [9], testing different ka around the FDA-reported values in their popPK models [8]. PK parameters were assumed to follow a log-normal distribution and, since both drugs are administered orally, they represent apparent values. Except for ka, IIV of each parameter was sequentially evaluated and integrated in the model if found to significantly improve data description. Different error models (i.e., additive, proportional and mixed) were tested to capture the residual unexplained variability using either the same random effects regardless of the analytical methods, or separate ones for each of them.

Biologically plausible factors were considered in the covariate analyses, including the following: liver function as classified by the National Cancer Institute Organ Dysfunction Working Group, Eastern Cooperative Oncology Group (ECOG) performance status, serum albumin, C-reactive protein (CRP), lactate dehydrogenase (LDH), sex, body weight, body mass index and age [17, 18]. Missing covariate values were not imputed. Covariate screening was conducted using an available-case approach, whereby records with missing values were excluded only from the corresponding covariate tests using the base model without the same records as reference model. Comedications susceptible to alter both drugs PK were also tested, such as cytochrome 3A4 (CYP3A4) inhibitors as described by [19] on encorafenib. Effect of hospitalization or ambulatory state were tested as well. Covariates (time-varying or not) were assessed for their potential influence on encorafenib and binimetinib PK using regressors in Monolix to model linear or allometric relationships as appropriate.

### Model selection and evaluation

The difference in the objective function value (ΔOFV), which approximates a Chi-square distribution, was used as a statistical discriminator between nested models. A significance level of 0.05 (ΔOFV < − 3.84 per one additional parameter) was applied during forward selection of the base model and identification of significant covariate-parameter relationship. A significance level of 0.01 (ΔOFV > 6.63 for the removal of one parameter) guided backward analysis to select covariate to retain in the final model. Diagnostic tools such as goodness-of-fit (GOF) plots and relative standard error (RSE) estimates of the parameters, along with their clinical relevance and general model stability, were as well considered in the model building steps.

Both final binimetinib and encorafenib popPK models underwent thorough internal validations. A bootstrap resampling procedure with 2000 replicates issued from the initial dataset was conducted to compare the original parameter estimates with the median and 95% confidence intervals (CI_95%_) of the bootstrap parameters for each drug. Prediction-corrected visual predictive checks (pcVPC) were performed using 1000 simulations of each final drug model to compare concentration-time profiles of the observed data with the 5th, 50th, and 95th prediction percentiles and their associated 90% CI [20].

### Model-based bayesian predictions in real-world patients and monte carlo simulations

Individual PK metrics (i.e., AUC_24h_ for encorafenib, AUC_12h_ for binimetinib, C_max_, C_min_, terminal t_1/2_ or T_max_, for both drugs at standard regimens) were predicted using the final model’s PK parameters and their respective IIV estimates, excluding the residual error. These PK metrics, summarized using geometric mean and coefficient of variation (CV%), were then descriptively compared with values reported in the literature, namely the Phase Ib/II (CMEK162X2110) and Phase III (COLUMBUS) studies conducted in unresectable melanoma patients, and the Phase II (PHAROS) study in metastatic non-small cell lung cancer [3, 8, 9, 21].

An exploratory investigation of the appropriateness of the PK metrics with in vitro derived PD parameters, as proposed by Goldstein et al. [11], was performed. The individual average concentration at steady state (C_average_) was obtained by dividing the AUC_τ_ by its respective dosing interval (τ), which is 24 h for encorafenib and 12 h for binimetinib. The unbound C_average_ were calculated by multiplying the steady state geometric mean of C_average_ in BRAF^V600E/K^-mutant metastatic melanoma by the unbound fraction, ${f}_{u}$ (0.1390 and 0.0280 for encorafenib and binimetinib, respectively), as illustrated below [11]:

$${Unbound\;C}_{average}={f}_{u}\cdot\frac{{AUC}_{{\uptau}}}{\tau}$$

The geometric mean of unbound C_average_ and C_min_ with their CV(%) were compared to each drug’s literature reported IC_50_, i.e., 2.16 ng/mL and 15.7 ng/mL for encorafenib and binimetinib, respectively [11].

Standard dosing regimen of binimetinib (45 mg BID) and encorafenib (450 mg QD) as well as a reduced dosing (300 mg QD) at steady state were simulated on 1000 virtual patients. This allowed retrieving median and 90% prediction interval between the 5th and 95th percentiles to construct real-world reference percentile curves. Following the same procedure, the geometric mean of unbound C_average_ and the corresponding unbound C_average_/IC_50_ ratios were computed for each simulated dosing schedule for each drug.

## Results

### Studied population

Table 1 summarizes the characteristics of the studied population. The popPK analysis was performed using a total of 310 concentrations of encorafenib from 66 patients and 311 concentrations of binimetinib from 69 patients with BRAF^V600E/K^-mutant metastatic melanoma. A median (range) of 3 [1–15] samples per patient, with one sample collected per occasion, were available for each drug. Median (range) time after last dose intake was 15.00 h (0.33–29.00) and 12.00 h (0.25–20.00) for encorafenib and binimetinib, respectively. An important proportion of concentrations were sampled at the end of dosing interval (24 h and 12 h), i.e. 28% for encorafenib and 63% for binimetinib, while only 6% and 7% of the concentrations of encrorafenib and binimetinib were collected before their respective T_max_ of 1.5 h and 1.1 h [1, 8, 9]. The median (range) dose was 450 (200–600) mg given once a day for encorafenib, and 45 mg (30–45) mg once to twice a day for binimetinib. Standard dosing of encorafenib (i.e., 450 mg QD) was administered in 81% of patients, while 300 mg QD was given to 12%. Dosing regimens of binimetinib were mainly standard dosing with 81% of patients receiving 45 mg and 19% 30 mg BID. Regarding the covariates, the highest proportion of missing data was observed for CRP, reaching up to 12% for encorafenib and 15% for binimetinib. Only 2 patients accounting for a total of 6 samples were simultaneously taking amiodarone, a moderate CYP3A4 inhibitor [19].

**Table 1:** Characteristics of the studied population

		Median (inter-quantile range) or *n* (%)	
Population characteristics at the baseline		Encorafenib	Binimetinib
Age [years]		62 (52–71)	62 (52–71)
Sex			
	Female	21 (32)	20 (29)
	Male	45 (68)	45 (65)
Weight [kg]		74.30 (65.00–87.00)	74.00 (64.80–87.00)
Body Mass Index [kg/m²]		25.01 (22.29–27.74)	24.78 (22.32–27.72)
Fat-Free Mass^a^ [kg]		48.34 (40.11–54.93)	48.01 (40.62–54.76)
Fat-Free Mass Index [kg/m²]		15.82 (14.52–17.36)	15.77 (14.37–17.39)
Bilirubin [µmol/L]		5.05 (4.00–8.00)	5.00 (3.65–7.00)
Aspartate Aminotransferase [UI/L]		25.80 (21.10–30.00)	26.00 (21.92–30.75)
Alanine Aminotransferase [UI/L]		22.00 (16.92–29.25)	23.00 (19.00–31.50)
Albumin [g/L]		39.70 (36.40–43.00)	39.70 (36.62–42.42)
Alkaline Phosphatase [UI/L]		67.50 (51.50–105.00)	71.00 (54.00–107.00)
Gamma-Glutamyl Transferase [UI/L]		44.50 (21.75–99.50)	45.00 (22.75–115.25)
Hepatic Function^b^			
	Normal	43 (65)	40 (58)
	Mild Dysfunction	16 (24)	16 (23)
Creatinine [µmol/L]		81.00 (68.00–91.50)	81.00 (70.50–91.50)
Creatinine Clearance [mL/min]		86.90 (65.36–100.06)	85.09 (64.70–99.04)
Estimated GFR^c^ [mL/min/1.73 m²]		75.29 (61.01–94.71)	70.09 (60.90–93.03)
	(≥ 90)	20 (30)	18 (26)
	(60–89)	31 (47)	31 (45)
	(30–59)	10 (15)	10 (14)
Lactate Dehydrogenase [U/L]		226.00 (189.00–271.00)	226.00 (187.00–284.00)
C-Reactive Protein [mg/L]		4.00 (2.00–7.45)	4.00 (2.00–9.55)
Lymphocyte^d^ Count [10^9^/L]		1.65 (1.11–2.16)	1.65 (1.08–2.12)
	No Lymphopenia	48 (73)	45 (65)
	Lymphopenia	13 (20)	13 (19)
Neutrophil^e^ Count [10^9^/L]		3.57 (2.65–4.86)	3.56 (2.73–5.35)
	No Neutropenia	59 (89)	56 (81)
	Mild Neutropenia	2 (3)	2 (3)
ECOG^f^			
	ECOG 0	39 (59)	36 (52)
	ECOG 1	23 (35)	21 (30)
	ECOG 2	1 (2)	1 (1)
	ECOG 3	1 (2)	2 (3)

^a^ Fat free mass as calculated by [24]

^b^ Hepatic function as described by the National Cancer Institute Organ Dysfunction Working Group [17]

^c^ Estimated Glomerular Filtration Rate based on the full age spectrum equation of [25]

^d^ Lymphopenia as defined by [26]

^e^ Neutropenia as defined by [27]

^f^ Eastern Cooperative Oncology Group Performance Status [18]

Percentages for categorical covariates may not sum to 100% because an ‘unknown/missing’ category is not displayed

### Population pharmacokinetic model

The data of both drugs were better described by a two-compartment model with linear elimination compared to a one compartment model (ΔOFV = −180, *p* < 0.001 for encorafenib and ΔOFV = −104, *p* < 0.001 for binimetinib). Ka was fixed at 0.51 h^−1^ for encorafenib and at 0.76 h^−1^ for binimetinib, corresponding to a Tmax of 1.5 h and 1.1 h, respectively, consistent with the literature [8, 9]. Inclusion of IIV on CL significantly improved the model for both drugs (ΔOFV = −84, *p* < 0.001 for binimetinib and ΔOFV = − 150, *p* < 0.001 for encorafenib). The addition of IIV on inter-compartmental clearance (Q) allowed a better fit of the encorafenib model (ΔOFV = −16, *p* < 0.01), which was further improved by inclusion of IIV on central volume of distribution (Vc) (ΔOFV = −11, *p* < 0.01). The introduction of IIV on Q in the binimetinib model, although not statistically significant (ΔOFV = + 6.53, *p* > 0.01), helped model stabilization and allowed appropriate estimation of the peripheral volume of distribution (Vp).

None of the studied covariates (tested on Vc, Q and CL for encorafenib, and on Q and CL for binimetinib), were significantly associated with the IIV of encorafenib or binimetinib.

Table 2 presents the estimates of the final popPK models and the results of non-parametric bootstrap analysis. The parameter estimations from the final model were consistent with their bootstrap counterparts for both encorafenib and binimetinib. For encorafenib, the differences between the final model and bootstrap estimates were less than 20%, with the bootstrap CI_95%_ of all the parameters encompassing the estimates from the final model. Similarly, for binimetinib, most parameters aligned well with bootstrap medians, except for Vp, Q and its IIV, which showed 69%, 57% and 137% difference, respectively. Yet, all of the final model estimates were included in the 95% CI of the bootstrap.

**Table 2:** Final population PK parameter estimates of encorafenib and binimetinib and bootstrap results

		Encorafenib			Binimetinib		
		Final Model		Bootstrap (*n* = 2000)	Final Model		Bootstrap (*n* = 2000)
Parameters		Estimate (RSE%)	Shrinkage (%)	Median (CI 95%)	Estimate (RSE%)	Shrinkage (%)	Median (CI 95%)
*CL [L/h]*		29 (5.0)	/	28 (22–35)	23 (4.2)	/	20 (9.7–24)
	*ω*_*Cl*_ *[CV%]*	18 (18)	32	17 (7–26)	23 (14)	21	23 (14–49)
*Vc [L]*		39 (16)	/	42 (11–67)	35 (23)	/	41 (0–80)
	*ω*_*Vc*_ *[CV%]*	71 (21)	38	62 (23–243)	/	/	/
*Q [L/h]*		1.7 (23.8)	/	1.6 (0.7–4.7)	35 (50)	/	22 (11–164)
	*ω*_*Q*_ *[CV%]*	92 (19)	25	77 (15–188)	168 (27)	27	71 (20–662)
*Vp [L]*		26 (12)	/	31 (14–540)	106 (10)	/	343 (84–2605)
*ka [h* ^*− 1*^ *]*		0.5 Fixed	/	0.5 Fixed	0.7 Fixed	/	0.7 Fixed
σ_prop_ *[CV%]*		47 (5.0)	/	46 (41–51)	46 (5.0)	/	46 (41–53)

**ka** : typical first-order absorption rate constant; **Vc** : typical apparent central volume of distribution; **Q**: typical apparent inter-compartmental clearance; **Vp**: typical apparent peripheral volume of distribution; **CL** : typical first order apparent clearance; **ω**: inter-individual variability parameter, **σ**_**prop**_ : proportional residual error, **CI 95%**: confidence interval between 2.5% and 97.5%; **RSE**: relative standard error. **CV** (%) for **ω** and **σ**_**prop**_ is calculated as follows:$CV=\sqrt{({e}^{{\omega}^{2}}-1)}$

**Fig. 1:**
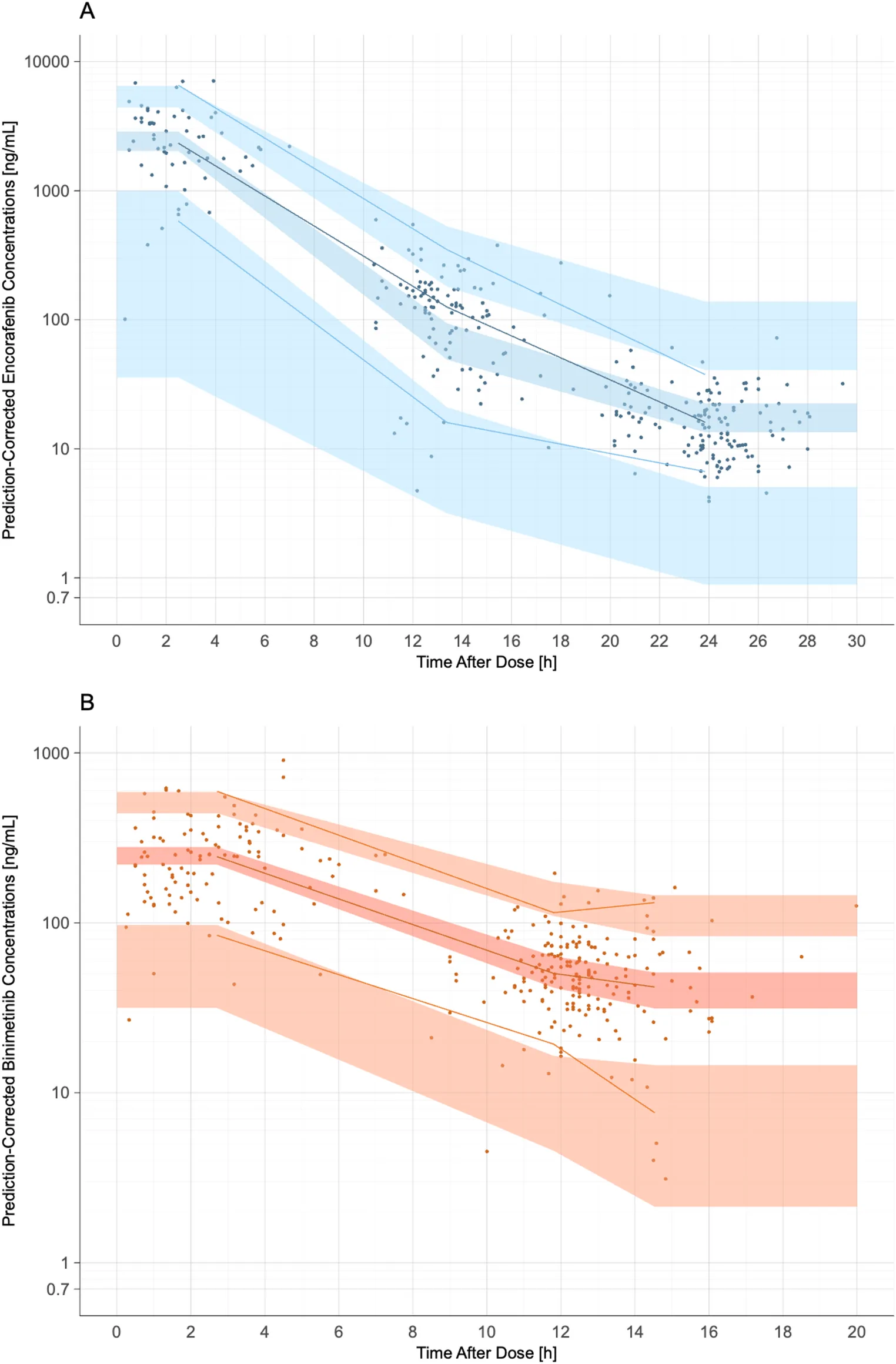
Encorafenib (**A**) and binimetinib (**B**) prediction-corrected visual predictive check (pcVPC) of the final model in log-transformed y axis. The points represent the prediction-corrected observed concentrations, the solid lines represent the 5th, 50th (median) and 95th percentiles of the observed concentrations. Shaded areas depict the 90% prediction intervals around the 5th, 50th (median) and 95th percentiles. PcVPCs were stratified into four bins defined by the following boundaries: 0.3, 8.1, 13.1, and 20.0 h for binimetinib, and 0.3, 8.7, 18.3, and 29.4 h for encorafenib

PcVPCs, employed to normalize concentrations given the heterogeneous dosing schedules observed in the population, supported adequate model performance for both drugs (Fig. 1). For encorafenib, the observed 5th and 95th percentiles largely fall within the simulated prediction intervals across time. A modest deviation is observed for the median between approximately 8 and 16 h, which trends slightly outside the simulated median band. Otherwise, central tendency and dispersion are well captured. For binimetinib, central tendency was well captured and the observed 5th and 95th percentiles generally fell within the simulated bands, apart from a small deviation of the 5th observed percentile.

The pcVPC, together with GOF, presented in Supplementary Fig. 1, and bootstrap results, allowed validation of the final models.

### Model-based bayesian predictions in real-world patients and monte carlo simulations

**Fig. 2:**
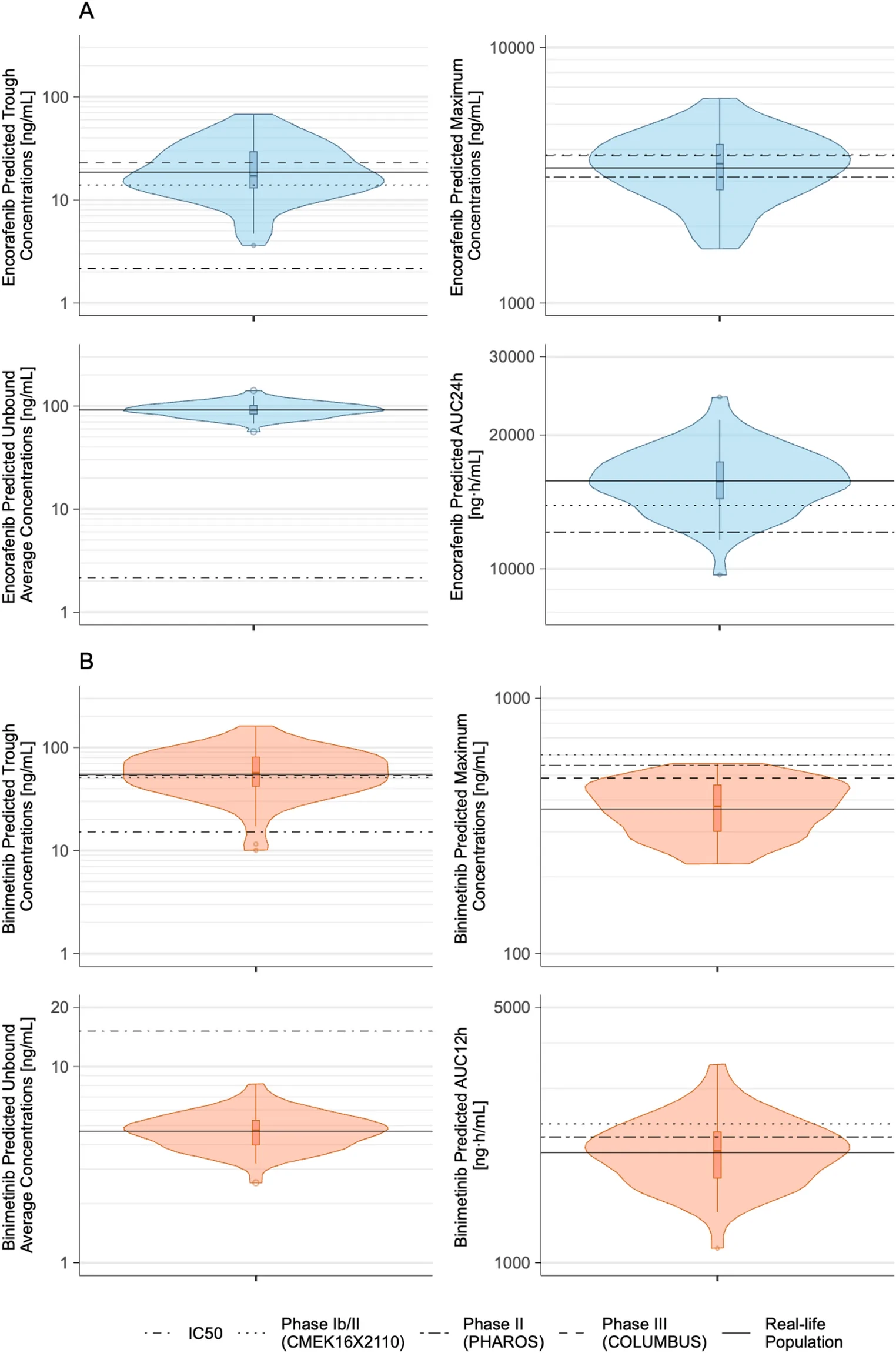
Encorafenib (**A**, blue) and binimetinib (**B**, orange) steady state exposure metrics based on respectively 61 and 53 real-life patients in comparison to in vitro IC_50_ of 2.16 and 15.17 ng/mL respectively (dashed/dotted line), and geometric means from clinical trials Phase I/IIB (dotted line) and III (dashed line) at standard dosing on log-transformed y axis [8, 9, 11]. The solid line represents the geometric mean of each exposure metric of the real-life patients. Boxplots represent median and interquantile range, violin the predicted metrics distribution and the unfilled dots the outliers of the real-life patients

Table 3; Fig. 2 compare the predicted PK metrics of individuals under standard regimen to those reported in Phase Ib/II, II, and III clinical trials [3, 8, 9]. In the real-world population, model-based Bayesian predictions revealed a geometric mean (CV%) C_min_ for encorafenib of 19 ng/mL (73%), while C_max_ was 3378 ng/mL (30%), and t_1/2_ was 12.5 h (95%). The geometric mean (CV%) of AUC_24h_ was estimated at 15,785 ng·h/mL (17%), and median (range) T_max_ was 1.5 (0.4–3.0) h. For binimetinib, the predicted geometric mean (CV%) of C_min_ was 55 ng/mL (56%), with a C_max_ of 367 ng/mL (24%), and a t_1/2_ of 7.3 (67%) hours. The geometric mean (CV%) AUC_12h_ was calculated at 2003 ng·h/mL (22%), with a T_max_ median (range) of 1.1 (0.8–2.3) h. Overall, PK metrics predicted in the real-world population were similar to those reported in clinical trials.

**Table 3:** Encorafenib and binimetinib steady state exposure metrics from the model-based Bayesian predictions in real-life population in comparison to literature references at standard dosing

	Geometric Mean (CV%) or Median [Range]									
	Encorafenib					Binimetinib				
Pharmacokinetic parameter	Real-life population (*n* = 66)	Phase Ib/II (CMEK162X2110) (*n* = 13)	Phase II (PHAROS) (*n* = 98)	Phase III (COLUMBUS) (*n* = 577)	EMA report	Real-life population (*n* = 69)	Phase Ib/II (CMEK162X2110) (*n* = 13)	Phase II (PHAROS) (*n* = 98)	Phase III (COLUMBUS) (*n* = 577)	EMA report
C_min_ [ng/mL]	18.6 (73)	13.9 (82)	/	23 (320)	/	55.0 (56)	51.4 (42)	/	53.3 (49)	/
C_max_ [ng/mL]	3378 (30)	3800 (63)	3110 (128.0)	3775 (70)	/	367 (24)	600 (39)	546 (61)	487 (27)	/
t_1/2_ [h]	12.5 (95)	3.5 (17)	/	4.1 (66)	12.1	7.3 (67)	3.5 (29)	/	4.7 (24)	8.7
AUC_τ_° [ng·h/mL]	15,785 (17)	13,900 (59)	12,100 (94.0)	16,716 (192)*	/	2003 (22)	2400 (59)	2210 (47)	2573 (36)*	/
T_max_ [h]	1.6 [0.4–3.0.4.0]	1.5 [0.5–2.6]	/	2 [0.5–2.0.5.0]	/	1.1 [0.8–2.3]	1.6 [0.5–2.6]	/	/	/

**C**_**min**_: trough concentrations (geometric mean); **C**_**max**_: maximal concentrations (geometric mean), t_1/2_: terminal half-life (geometric mean), **T**_**max**_: time to maximum concentration (median). Geometric means of the real-life population were calculated according to: $Geometric\;Mean=\mathrm{e}\mathrm{x}\mathrm{p}\left[\frac{1}{n}{\sum}log\left({X}_{i}\right)\right]$

°Area Under the Curve (AUC) over 24 h–12 h for respectively enorafenib and binimetinib, except for *, standing for AUC infinity (AUC_inf_). Only AUC_inf_ was reported at steady state for combination of encorafenib and binimetinib at standard dosing in the Phase III study. In this same report, no T_max_ was available for binimetinib. Likewise, Phase II PHAROS study only reported C_max_ and AUC_τ_

**Fig. 3:**
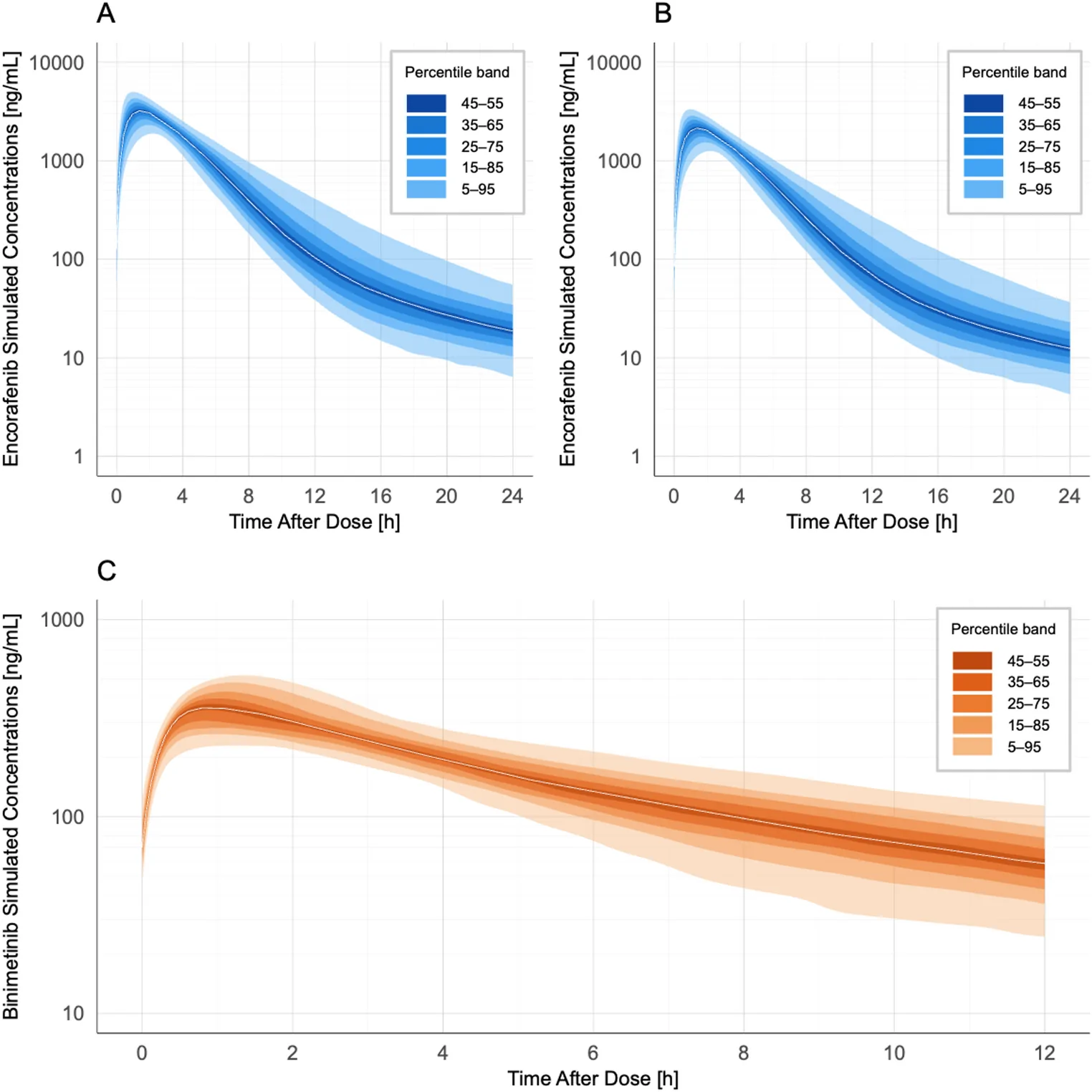
Simulations of encorafenib and binimetinib plasma concentrations at steady state for once daily 450 mg (**A**, blue), and 300 mg (**B**, blue) encorafenib or 45 mg binimetinib twice a day (**C**, orange). The solid white line represents the median (50th percentile). The light and dark shaded areas cover the 90% prediction interval

Using patients predictions of unbound C_average_ of 68.74 and 4.67 ng/mL, and considering IC_50_ of 2.16 ng/mL and 15.7 ng/mL for encorafenib and binimetinib, respectively [11], the studied population exhibited an unbound C_average_/IC_50_ of 31.82 for encorafenib and 0.30 for binimetinib. Of note, encorafenib unbound C_average_ markedly exceeds its IC_50_, whereas binimetinib unbound C_average_ is distinctly below its IC_50_. Figure 3 shows Monte Carlo simulations of steady-state plasma concentrations for both encorafenib (450 or 300 mg QD) and binimetinib at standard dosing. At steady state, the simulated geometric mean unbound C_average_ and corresponding unbound C_average_/IC_50_ ratios were 59.8 ng/mL and 27.7 for encorafenib 300 mg QD, 89.7 ng/mL and 41.5 for encorafenib 450 mg QD, and 11.4 ng/mL and 0.75 for binimetinib 45 mg BID, respectively.

## Discussion

This study reports popPK models for encorafenib and binimetinib based on data from real-world patients with unresectable or metastatic melanoma with a BRAF^V600E/K^ mutation. Comparison of PK metrics with data from clinical trials support comparable plasma exposure and inter-individual variability, with no significant covariates identified in this population.

Concentration-time data were successfully described by a two-compartment model with first-order absorption and linear elimination for both drugs, in accordance with the manufacturer’s models [8]. However, while the manufacturer’s models used an autoinduction process to depict the elimination of both drugs, our analysis found that constant CL satisfactorily captured their elimination. This discrepancy can be explained by the fact that the samples were collected at steady state in the real-world population when autoinduction had already been achieved for both drugs [8]. The manufacturer popPK models described drugs absorption with a first-order process and a lag-time. However, limited data immediately after drugs intake prevented us from estimating absorption delays and ka values. We therefore decided to use a simple first-order absorption model and to fix ka values to achieve T_max_ values consistent with literature ranges [1, 8, 9]. The mean (CV%) model-derived elimination half-lives were 12.5 h (95%) and 7.3 h (67%), for encorafenib and binimetinib, respectively. These values are consistent with the manufacturer’s popPK model of encorafenib (12.1 h [5]), and the median half-life of 8.7 h reported for binimetinib [6]. The IIV on CL of encorafenib and binimetinib was low to moderate (18% and 23%, respectively) and no covariates were significantly associated with IIV on CL or other PK parameters in our study. In the FDA report, none of the studied covariates, including age, sex, body weight, or mild hepatic impairment, were found to significantly impact the PK of encorafenib [8]. Co-administration of encorafenib 50 mg (single dose) with repeated posaconazole, a strong CYP3A4 inhibitor, led to a 2.8-fold increase in AUC_inf_, as predicted by a physiologically-based pharmacokinetic (PBPK) model [8]. Similarly, administration of diltiazem, a moderate CYP3A4 inhibitor, resulted in a 1.8-fold increase in encorafenib AUC_inf_ [8]. These effects were deemed clinically significant, leading to a recommended dose reduction of encorafenib by two-thirds when co-administered with strong inhibitors and by half with moderate inhibitors [8]. Yet, due to the absence of patients taking strong CYP3A4 inhibitors and limited number of samples (*n* = 6) and patients (*n* = 2) taking moderate CYP3A4 inhibitors, their effect on encorafenib exposure could not be corroborated in our popPK analysis. Concerning binimetinib, its CL was reported to be influenced by both body weight and hepatic function [8]. No effect of the liver function during the covariate analysis of binimetinib was found, probably due to the absence of patients with moderate or severe hepatic dysfunction. The absence of patients with extreme covariate values in the studied real-world population as well as the limited number of patients could explain the difficulty to identify significant covariates in our analysis.

For both drugs, the distributions of individual-predicted PK metrics included the values reported in clinical trials, with the minor exception of the C_max_ distribution for binimetinib, which fell slightly below the Phase Ib/II geometric mean. The real-world population t_1/2_ and inter-individual variability were comparable with Phase Ib/II and III studies. The minor differences in C_max_, despite being within a comparable range, can be explained by the previously mentioned limited availability of data during the absorption phase.

No important differences concerning the ratio of unbound C_average_/IC_50_ were observed with those reported by Goldstein et al. [11], although the distributions of unbound C_average_ for both drugs did not encompass their respective IC_50_. Despite being subject to several limitations and assumptions, this approach allows a rough evaluation of the PK/PD relationship of encorafenib and binimetinib with respect to other kinase inhibitors, especially considering the lack of PK/PD studies [7, 11]. By computing the ratio of unbound C_average_ to the IC_50_ reported in the literature, Goldstein et al. found that most BRAF and MEK inhibitors exhibit a C_average_/IC_50_ ratio near 1, while the ratios for encorafenib, vemurafenib, and binimetinib deviate considerably from this value [11]. Notably, the elevated ratio of 41.5 for encorafenib (450 mg QD) suggests that lower doses might achieve therapeutic efficacy with a reduced risk of toxicity, whereas the lower ratio of 0.75 for binimetinib suggests that its exposure may fall significantly below its IC_50_. Future PK/PD studies are essential to better characterize the relationship between exposure, response, and efficacy for both drugs, using clinical endpoints such as PFS and ORR. In the absence of these PK/PD data, dose adjustments aimed at optimizing efficacy in routine clinical practice cannot be supported.

Safety data from phase III COLUMBUS trial show a high burden of toxicity and frequent dose modifications with encorafenib-containing regimens: grade 3/4 adverse events occurred in ~ 70% of patients, dose adjustments or interruptions were required in approximately 56.8% on encorafenib plus binimetinib (and 72.9% on encorafenib monotherapy), and treatment discontinuations due to adverse events reached ~ 20% on the combination. Though real-world evidence indicates a lower incidence of severe toxicity compared to pivotal trials, adverse events remain a significant clinical burden, affecting 42.7% of patients [22]. Notably, toxicity management frequently necessitates empirical dose modifications. In a recent cohort, 17.1% of patients required encorafenib dose reduction from 450 mg to 300 mg QD [22]. While these data do not establish causality between exposure and toxicity, they are compatible with the hypothesis that a subset of patients may experience clinically relevant over-exposure to encorafenib [23]. This reinforces the need for thorough and in-depth exposure–toxicity analyses to complement existing regulatory reports. The SPEBINI trial (NCT06774989) is currently ongoing in France to address these questions. The main objective is to assess the association between plasma exposure of encorafenib and binimetinib and the occurrence of dose-limiting toxicity during the first 3 months of treatment. As a secondary objective, the exposure-efficacy relationship (PFS, OS) will be assessed.

We acknowledge that single-sample occasions, sparse data, and occasional inaccuracies in patient-reported drug intake timing, associated with the retrospective nature of the study, represent major limitations of this analysis. This contributed to the need to fix the ka values as well as an unexplained residual error of approximately 50% in both models, which we deemed acceptable within the context of the study. Regarding encorafenib, visual diagnostics (pcVPC and NPDE) suggested a model misspecification, with observed concentrations transiently exceeding the prediction interval and appearing to follow a one-compartment profile. We attribute this visual deviation to data sparsity in the distribution phase rather than a structural deficiency, a conclusion supported by the wider confidence intervals observed in the bootstrap analysis for CIs of the parameters. Despite this limitation, the two-compartment structure was retained as the most appropriate model, supported by a substantial statistical improvement over the one-compartment model and consistency with the manufacturer’s reported model [8]. Similarly, differences between bootstrap and final estimates for binimetinib (Q, Vp, and their IIV) likely reflect limited information in the distribution phase and sensitivity to the inclusion of IIV on Q. Subject resampling in the nonparametric bootstrap may omit individuals who are informative for the distribution phase, thereby widening CIs and slightly shifting median estimates, while overall diagnostics (GOF, pcVPC) and exposure metrics remain adequate. Despite these limitations, the PK profiles of encorafenib and binimetinib observed in the studied population broadly align with data from clinical trials at standard dosing of each drug, supporting the validity of the real-world population findings.

## Conclusion

We present the first popPK model of encorafenib and binimetinib developed in a real-world clinical setting. Both drugs exhibited moderate inter-individual variability but no significant covariates explaining this variability were identified. Nevertheless, these popPK models may serve as a valuable foundation for future PK/PD studies investigating the relationship between drug exposure, efficacy, and toxicity.

## Supplementary Information

Below is the link to the electronic supplementary material.

Supplementary Material 1

## Data Availability

The data underlying this study contain sensitive participant information and are not publicly accessible; however, they can be obtained from the corresponding author (MG) upon reasonable request.
