# Supplementary material for: Population pharmacokinetics of encorafenib and binimetinib in real-world patients with BRAFV600E/K-mutant metastatic melanoma
Source: Cancer Chemother Pharmacol. 2026 Mar 17;96(1):29. doi: 10.1007/s00280-026-04876-y (PMC13661740; doi:10.1007/s00280-026-04876-y)
Supplement: Supplementary file 1 [file 280_2026_4876_MOESM1_ESM.docx]

# 1. Supplemental Material

## Supplemental Material 1: Determination of encorafenib and binimetinib concentrations in plasma

Quantification of encorafenib and binimetinib plasma concentrations was performed using a validated liquid chromatography method coupled with mass spectrometry detection (LC-MS/MS). Briefly, 200 µL of plasma (calibrator, internal quality control or patient sample) were spiked with 20 µL of a 70%/30% (v/v) mixture of methanol and dimethyl sulfoxide (DMSO) containing the internal standards (I.S., ^13^C, ^2^H_3_-encorafenib at 1000 ng/mL and ^13^C_2_, ^2^H_4_-binimetinib at 3000 ng/mL). After 5-second vortexing, 600 µL of acetonitrile with 1% (v/v) formic acid were added for protein precipitation. The mixture was vortexed for 5 minutes and centrifuged at 13,000 g for 10 minutes at room temperature. Forty microliters of supernatant were collected, then mixed with 160 µL of water with 0.1% (v/v) formic acid. After 10-second vortexing, the final mixture was transferred into prolypropylene vials, and then 20 µL were injected into a Vanquish® UHPLC system (ThermoFisher Scientific, Les Ulis, France). Chromatographic separation was performed on Accucore® C18 (2.1 x 50 mm; 2.6 µm) analytical column (ThermoFisher Scientific) associated with a guard column packed with the same bonded phase. The column temperature was maintained at 30°C and the auto-sampler at 4°C. The mobile phase consisted of a mixture of reagent A (water, 0.1% formic acid) and reagent B (acetonitrile, 0.1% formic acid) delivered at a flow rate of 500 µL/min in a gradient elution mode. The gradient started at 95% of reagent A, then changed gradually to achieve 65% of reagent B at 4 minutes. The gradient increased to 95% of reagent B from 4.0 to 4.5 minutes. Between 4.5 and 5 minutes, the conditions were maintained at 5:95 (A:B,%), and then went back to 95% reagent A at 5.1 minutes. Finally, the column was reconditioned at the starting conditions of 95:5 (A:B, %) for up to 3 min prior to the next injection. The retention times of encorafenib and its I.S. were 2.67 and 2.66 minutes, respectively, whereas those of binimetinib and its I.S. were 2.36 and 2.37, respectively. A TSQ Quantis® triple quadrupole mass spectrometer equipped with a Heated Electrospray Ionization (HESI) Probe (ThermoFisher Scientific) was used for mass detection and quantification. The instrument was operated in positive ion mode and the [M+H]+ ions. Encorafenib, binimetinib and their respective I.S. were quantified in selected reaction monitoring mode using the following transitions: m/z 541.2→372.2 for encorafenib, 545.2→513.2 for I.S. of encorafenib, 443.1→165.1 for binimetinib, and 449.1→165.2 for I.S. of binimetinib. The method was validated according to European Medicines Agency (EMA) guidelines for bioanalytical method validation. The calibration curve comprised standards ranging from 1 to 1000 ng/mL for both drugs and was quadratic for encorafenib and linear for binimetinib. The intra and inter-precision for 3 internal quality controls (15, 75 and 300 ng/mL) were below 9.6% and 10.7%, respectively, for encorafenib, and below 8.7% and 9.3% for binimetinib. The intra and inter-accuracy ranged from 93.6 to 107.1% for encorafenib and from 91.2% to 110.7% for binimetinib.

**Figure S1**. Goodness of fit plots of encorafenib (blue) and binimetinib (orange). Panel A shows the observed versus individual predicted concentrations with the line of identity (solid line) included for reference. In all panels, the solid red curve represents the LOESS smooth and the shaded ribbon its 95% confidence interval. Panel B displays population predicted versus observed concentrations. Panels C and D illustrate individual weighted residuals (IWRES) and E and F, normalized predicted errors (NPDE) plotted against both individual-predicted concentrations and time after dose, respectively.
